# Bis[2-(4-hy­droxy­phen­yl)acetato-κ*O*]bis­(1,10-phenanthroline-κ^2^ *N*,*N*′)cadmium penta­hydrate

**DOI:** 10.1107/S1600536811001747

**Published:** 2011-01-22

**Authors:** Yu-Ye Yu

**Affiliations:** a Jinhua College of Vocation and Technology, Jinhua, Zhejiang 321017, People’s Republic of China

## Abstract

In the title compound, [Cd(C_8_H_7_O_3_)_2_(C_12_H_8_N_2_)_2_]·5H_2_O, the Cd^II^ ion is six-coordinated by two carboxylate O atoms of monodentate 2-(4-hy­droxy­phen­yl)acetate ligands and by four N atoms from two chelating 1,10-phenantroline ligands in a distorted trigonal–prismatic geometry. O—H⋯O hydrogen bonds between water mol­ecules and the complex mol­ecules result in the formation of a three-dimensional network. Four water mol­ecules act as single acceptors and double donors while the fifth water mol­ecule is involved as a single acceptor and single donor in an O—H⋯O inter­action and as a donor in an O—H⋯π inter­action.

## Related literature

For metal complexes derived from carb­oxy­lic acids, see: Fang & Zhang (2006 ▶); Pan *et al.* (2006 ▶); Wang & Sevov (2008 ▶); Wang *et al.* (2010 ▶); Liu *et al.* (2010 ▶).
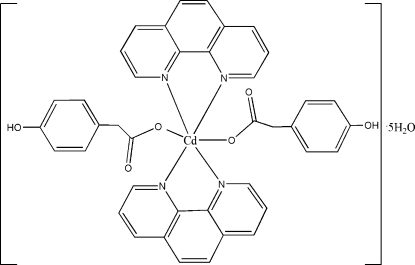


## Experimental

### 

#### Crystal data

[Cd(C_8_H_7_O_3_)_2_(C_12_H_8_N_2_)_2_]·5H_2_O
*M* *_r_* = 865.17
Triclinic, 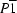

*a* = 11.020 (1) Å
*b* = 11.341 (1) Å
*c* = 16.554 (2) Å
α = 86.170 (1)°
β = 77.537 (1)°
γ = 70.836 (1)°
*V* = 1908.2 (3) Å^3^
*Z* = 2
Mo *K*α radiation
μ = 0.64 mm^−1^
*T* = 296 K
0.31 × 0.29 × 0.11 mm

#### Data collection

Bruker APEXII area-detector diffractometer
Absorption correction: multi-scan (*SADABS*; Sheldrick, 1996 ▶) *T*_min_ = 0.823, *T*_max_ = 0.930
28812 measured reflections
8705 independent reflections
7876 reflections with *I* > 2σ(*I*)
*R*_int_ = 0.020

#### Refinement

*R*[*F* ^2^ > 2σ(*F* ^2^)] = 0.026
*wR*(*F* ^2^) = 0.067
*S* = 1.04
8705 reflections
505 parameters
7 restraints
H-atom parameters constrained
Δρ_max_ = 0.60 e Å^−3^
Δρ_min_ = −0.30 e Å^−3^

### 

Data collection: *APEX2* (Bruker, 2006 ▶); cell refinement: *SAINT* (Bruker, 2006 ▶); data reduction: *SAINT*; program(s) used to solve structure: *SHELXS97* (Sheldrick, 2008 ▶); program(s) used to refine structure: *SHELXL97* (Sheldrick, 2008 ▶); molecular graphics: *SHELXTL* (Sheldrick, 2008 ▶); software used to prepare material for publication: *SHELXTL*.

## Supplementary Material

Crystal structure: contains datablocks I, global. DOI: 10.1107/S1600536811001747/gk2315sup1.cif

Structure factors: contains datablocks I. DOI: 10.1107/S1600536811001747/gk2315Isup2.hkl

Additional supplementary materials:  crystallographic information; 3D view; checkCIF report

## Figures and Tables

**Table 1:** Hydrogen-bond geometry (Å, °) *Cg*5 is the centroid of the C3–C8 ring.

*D*—H⋯*A*	*D*—H	H⋯*A*	*D*⋯*A*	*D*—H⋯*A*
O3—H3*A*⋯O4^i^	0.82	1.82	2.641 (2)	175
O6—H6*A*⋯O4*W*^ii^	0.82	1.87	2.670 (3)	164
O1*W*—H1*WA*⋯O5^iii^	0.83	1.93	2.756 (2)	177
O1*W*—H1*WB*⋯O6^ii^	0.77	2.03	2.798 (3)	177
O2*W*—H2*WA*⋯O1^iv^	0.81	2.01	2.812 (2)	168
O2*W*—H2*WB*⋯O1*W*	0.76	2.04	2.769 (3)	160
O3*W*—H3*WA*⋯O3^i^	0.84	2.02	2.817 (3)	158
O3*W*—H3*WB*⋯O2*W*	0.82	1.94	2.712 (3)	155
O4*W*—H4*WB*⋯O5*W*	0.87	1.82	2.682 (3)	168
O5*W*—H5*WB*⋯O2	0.84	1.92	2.756 (2)	176
O5*W*—H5*WA*⋯O3*W*	0.92	1.87	2.737 (3)	157
O4*W*—H4*WA*⋯*Cg*5^v^	0.82	2.85	3.583 (2)	151

Symmetry codes: (i) 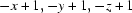; (ii) 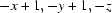; (iii) 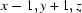; (iv) 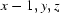; (v) 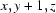.

**Fig. 1.**
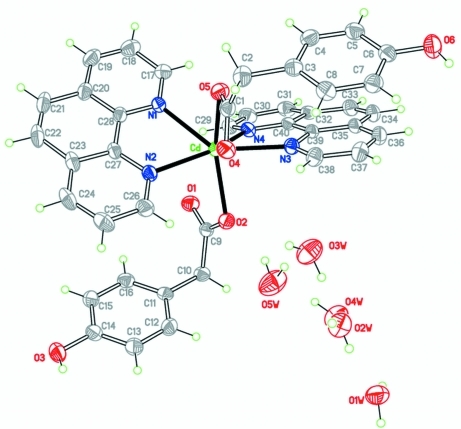
The molecular structure of the title complex. Displacement ellipsoids are drawn at the 30% probability level.

**Fig. 2.**
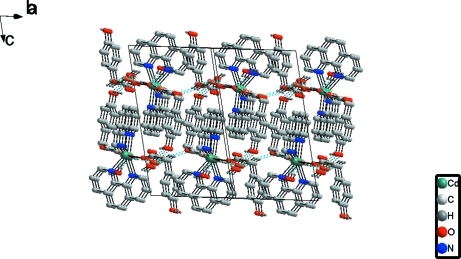
The crystal packing of the complex.

## supplementary crystallographic information

### Comment

The design and synthesis of carboxylic metal-organic complexes have been of increasing interest for decades owing to their potential practical applications including fluorescence and magnetism (Wang, *et al.*, 2010; Fang, *et al.*, 2006; Wang, *et al.*, 2008). We have worked at it before (Liu, *et al.*, 2010). In the paper, we report the crystal structure of a new cadmiun(II) complex with *p*- hydroxyphenylacetic acid and 1,10-phenanthroline.

The structure of the complex is shown in Fig.1, which shows that the Cd(II) atom is coordinated by two *p*-hydroxyphenylacetate(PAA) anions and two 1,10-phenanthroline (phen)ligands. The monodentate PAA anions coordinate to the Cd(II) ion in an approximate *trans* configuration, their benzene rings being nearly parallel to each other. The phen acts as a chelate ligand *via* the N atoms, while the carboxylate ligand has one carboxylate groups,behaving as a monodentate site through the deprotonated O atom. The coordination geometry can be described as a distorted trigonal prism. The coordination compound is built up by a pair of PAA anions using carboxylate oxygen atoms (Cd—O2=2.3222 (15) Å,Cd—O5=2.3676 (16) Å) and by a pair of neutral 1,10-phenanthroline molecules using nitrogen atoms(Cd—N1= 2.4522 (17) Å,Cd—N2=2.3577 (16) Å,Cd—N3=2.4472 (17) Å,Cd—N4=2.3786 (18) Å)in *trans* positions(Pan, *et al.*, 2006).

The packing plot is shown in Fig.2. The most significant forces contribulting the formation and stabilization of the crystal are O—H···O hydrogen bonds and weak π···π aromatic interactions between phen molecules and aromatic rings of the carboxylate ligands.

### Experimental

All reagents were of analytical grade and were used without further purification. 4-Hydroxyphenylacetic acid (0.152 g, 1 mmol) and 1,10- phenanthroline (0.1982 g, 1 mmol) were added to a solution of Cd(OH)_2_ (0.146 g,1 mmol) in 10 ml e thanol. The solution was stirred at 343 K for 12 days, and then 10 ml of ethanol were added. A wite deposit was formed within a few minutes that was kept for 12 days at 313 K. The deposit was filtered off and colorless solution was slowly evaporated resulting in formation of colorless single crystals of the title compound within 5 days.

### Refinement

The H atoms bonded to C atoms were positioned geometrically and refined using a riding model approximation [*C*—*H*(methylene)=0.97 Å, *U*_iso_(H) = 1.2*U*_eq_(C); aromatic C—H = 0.93 Å,*U*_iso_(H) = 1.2 *U*_eq_(C)]. Water and hydroxylic H atoms were located in difference Fourier maps and refined as riding on their carrier atoms with *U*_iso_(H) = 1.5*U*_eq_(O). Seven rigid-bond restraints to *U^ij^*-values of Co and the coordinating O and N atoms were imposed *via SHELXL97* DELU instructions.

### Crystal data

**Table:**

[Cd(C_8_H_7_O_3_)_2_(C_12_H_8_N_2_)_2_]·5H_2_O	*Z* = 2
*M**_r_* = 865.17	*F*(000) = 888
Triclinic, *P*1	*D*_x_ = 1.506 Mg m^−^^3^
Hall symbol: -P 1	Mo *K*α radiation, λ = 0.71073 Å
*a* = 11.020 (1) Å	Cell parameters from 9977 reflections
*b* = 11.341 (1) Å	θ = 1.3–27.7°
*c* = 16.554 (2) Å	µ = 0.64 mm^−^^1^
α = 86.170 (1)°	*T* = 296 K
β = 77.537 (1)°	Block, colourless
γ = 70.836 (1)°	0.31 × 0.29 × 0.11 mm
*V* = 1908.2 (3) Å^3^	

### Data collection

**Table:**

Bruker APEXII area-detector diffractometer	8705 independent reflections
Radiation source: fine-focus sealed tube	7876 reflections with *I* > 2σ(*I*)
graphite	*R*_int_ = 0.020
φ and ω scans	θ_max_ = 27.7°, θ_min_ = 1.3°
Absorption correction: multi-scan (*SADABS*; Sheldrick, 1996)	*h* = −14→14
*T*_min_ = 0.823, *T*_max_ = 0.930	*k* = −14→14
28812 measured reflections	*l* = −21→21

### Refinement

**Table:**

Refinement on *F*^2^	Primary atom site location: structure-invariant direct methods
Least-squares matrix: full	Secondary atom site location: difference Fourier map
*R*[*F*^2^ > 2σ(*F*^2^)] = 0.026	Hydrogen site location: inferred from neighbouring sites
*wR*(*F*^2^) = 0.067	H-atom parameters constrained
*S* = 1.04	*w* = 1/[σ^2^(*F*_o_^2^) + (0.0315*P*)^2^ + 0.6164*P*] where *P* = (*F*_o_^2^ + 2*F*_c_^2^)/3
8705 reflections	(Δ/σ)_max_ = 0.002
505 parameters	Δρ_max_ = 0.60 e Å^−^^3^
7 restraints	Δρ_min_ = −0.30 e Å^−^^3^

### Special details

**Table:**

Geometry. All e.s.d.'s (except the e.s.d. in the dihedral angle between two l.s. planes) are estimated using the full covariance matrix. The cell e.s.d.'s are taken into account individually in the estimation of e.s.d.'s in distances, angles and torsion angles; correlations between e.s.d.'s in cell parameters are only used when they are defined by crystal symmetry. An approximate (isotropic) treatment of cell e.s.d.'s is used for estimating e.s.d.'s involving l.s. planes.
Refinement. Refinement of *F*^2^ against ALL reflections. The weighted *R*-factor *wR* and goodness of fit *S* are based on *F*^2^, conventional *R*-factors *R* are based on *F*, with *F* set to zero for negative *F*^2^. The threshold expression of *F*^2^ > σ(*F*^2^) is used only for calculating *R*-factors(gt) *etc*. and is not relevant to the choice of reflections for refinement. *R*-factors based on *F*^2^ are statistically about twice as large as those based on *F*, and *R*- factors based on ALL data will be even larger.

### Fractional atomic coordinates and isotropic or equivalent isotropic displacement parameters (Å^2^)

**Table:**

	*x*	*y*	*z*	*U*_iso_*/*U*_eq_	
Cd	0.921272 (12)	0.297983 (12)	0.262351 (7)	0.03811 (5)	
N1	1.09784 (15)	0.16075 (14)	0.32637 (9)	0.0420 (3)	
N2	0.85159 (15)	0.29728 (14)	0.40757 (9)	0.0429 (3)	
N3	0.85430 (16)	0.32506 (15)	0.12900 (9)	0.0434 (3)	
N4	1.09251 (15)	0.32116 (15)	0.15377 (9)	0.0437 (3)	
O1	0.98347 (13)	0.49440 (13)	0.30979 (9)	0.0518 (3)	
O1W	0.09649 (18)	0.88079 (17)	0.19886 (11)	0.0813 (5)	
H1WA	0.0371	0.9464	0.2152	0.122*	
H1WB	0.1537	0.8990	0.1722	0.122*	
O2	0.79307 (13)	0.50615 (12)	0.28232 (8)	0.0479 (3)	
O2W	0.15247 (16)	0.63322 (18)	0.24663 (11)	0.0727 (4)	
H2WA	0.1051	0.5953	0.2718	0.109*	
H2WB	0.1219	0.7036	0.2420	0.109*	
O3	0.56429 (16)	0.71568 (16)	0.67907 (9)	0.0653 (4)	
H3A	0.4843	0.7357	0.6849	0.098*	
O3W	0.41174 (18)	0.5009 (2)	0.22350 (13)	0.0895 (6)	
H3WA	0.4111	0.4504	0.2630	0.134*	
H3WB	0.3415	0.5578	0.2335	0.134*	
O4	0.69434 (15)	0.21911 (14)	0.29253 (9)	0.0568 (4)	
O4W	0.52550 (19)	0.82720 (19)	0.14562 (13)	0.0908 (6)	
H4WA	0.5898	0.8509	0.1342	0.136*	
H4WB	0.5441	0.7687	0.1822	0.136*	
O5	0.89776 (14)	0.10086 (13)	0.24775 (9)	0.0507 (3)	
O5W	0.5459 (2)	0.6477 (2)	0.26075 (17)	0.1118 (8)	
H5WB	0.6203	0.6061	0.2698	0.168*	
H5WA	0.5178	0.5820	0.2545	0.168*	
O6	0.69850 (16)	0.05348 (17)	−0.09615 (9)	0.0695 (4)	
H6A	0.6235	0.0923	−0.1015	0.104*	
C1	0.7764 (2)	0.11842 (18)	0.26384 (11)	0.0443 (4)	
C2	0.7300 (2)	0.0149 (2)	0.24301 (13)	0.0556 (5)	
H2A	0.6451	0.0219	0.2780	0.067*	
H2B	0.7914	−0.0653	0.2534	0.067*	
C3	0.7190 (2)	0.02282 (18)	0.15278 (12)	0.0471 (4)	
C4	0.8299 (2)	−0.02101 (19)	0.09063 (14)	0.0544 (5)	
H4A	0.9111	−0.0584	0.1049	0.065*	
C5	0.8224 (2)	−0.0104 (2)	0.00855 (14)	0.0560 (5)	
H5A	0.8982	−0.0395	−0.0320	0.067*	
C6	0.7025 (2)	0.04348 (19)	−0.01398 (12)	0.0495 (4)	
C7	0.5906 (2)	0.0863 (2)	0.04668 (13)	0.0524 (5)	
H7A	0.5092	0.1215	0.0322	0.063*	
C8	0.5997 (2)	0.0769 (2)	0.12918 (13)	0.0530 (5)	
H8A	0.5240	0.1076	0.1696	0.064*	
C9	0.86740 (18)	0.55353 (17)	0.30747 (10)	0.0404 (4)	
C10	0.8083 (2)	0.68753 (17)	0.33848 (12)	0.0457 (4)	
H10A	0.8769	0.7257	0.3308	0.055*	
H10B	0.7444	0.7344	0.3063	0.055*	
C11	0.74249 (18)	0.69351 (16)	0.42914 (11)	0.0413 (4)	
C12	0.6094 (2)	0.7498 (2)	0.45409 (13)	0.0591 (5)	
H12A	0.5593	0.7836	0.4145	0.071*	
C13	0.5483 (2)	0.7572 (2)	0.53676 (13)	0.0637 (6)	
H13A	0.4580	0.7957	0.5521	0.076*	
C14	0.6205 (2)	0.70798 (19)	0.59631 (12)	0.0490 (4)	
C15	0.7538 (2)	0.6497 (2)	0.57256 (12)	0.0509 (5)	
H15A	0.8035	0.6150	0.6122	0.061*	
C16	0.81361 (19)	0.64290 (19)	0.48983 (12)	0.0485 (4)	
H16A	0.9037	0.6034	0.4745	0.058*	
C17	1.2147 (2)	0.08956 (19)	0.28732 (13)	0.0528 (5)	
H17A	1.2354	0.0929	0.2299	0.063*	
C18	1.3081 (2)	0.0099 (2)	0.32820 (16)	0.0655 (6)	
H18A	1.3892	−0.0384	0.2986	0.079*	
C19	1.2784 (2)	0.0042 (2)	0.41199 (16)	0.0646 (6)	
H19A	1.3405	−0.0468	0.4403	0.078*	
C20	1.1556 (2)	0.07441 (19)	0.45566 (13)	0.0513 (5)	
C21	1.1149 (3)	0.0674 (2)	0.54353 (14)	0.0646 (6)	
H21A	1.1735	0.0156	0.5738	0.078*	
C22	0.9952 (3)	0.1335 (2)	0.58284 (13)	0.0649 (6)	
H22A	0.9717	0.1263	0.6399	0.078*	
C23	0.9022 (2)	0.2150 (2)	0.53929 (11)	0.0522 (5)	
C24	0.7749 (3)	0.2854 (2)	0.57780 (13)	0.0643 (6)	
H24A	0.7481	0.2820	0.6349	0.077*	
C25	0.6904 (2)	0.3586 (2)	0.53231 (14)	0.0646 (6)	
H25A	0.6058	0.4057	0.5577	0.078*	
C26	0.7324 (2)	0.3620 (2)	0.44643 (13)	0.0551 (5)	
H26A	0.6739	0.4119	0.4154	0.066*	
C27	0.93710 (19)	0.22480 (17)	0.45242 (10)	0.0414 (4)	
C28	1.06665 (19)	0.15289 (16)	0.40979 (11)	0.0406 (4)	
C29	1.2023 (2)	0.3332 (2)	0.16604 (13)	0.0524 (5)	
H29A	1.2139	0.3319	0.2201	0.063*	
C30	1.3013 (2)	0.3478 (2)	0.10237 (14)	0.0591 (5)	
H30A	1.3773	0.3558	0.1137	0.071*	
C31	1.2851 (2)	0.3500 (2)	0.02283 (14)	0.0583 (5)	
H31A	1.3513	0.3574	−0.0207	0.070*	
C32	1.1692 (2)	0.34120 (18)	0.00681 (11)	0.0480 (4)	
C33	1.1421 (2)	0.3504 (2)	−0.07470 (12)	0.0585 (6)	
H33A	1.2055	0.3587	−0.1199	0.070*	
C34	1.0270 (2)	0.3472 (2)	−0.08689 (12)	0.0576 (5)	
H34A	1.0125	0.3522	−0.1405	0.069*	
C35	0.9262 (2)	0.33649 (17)	−0.01936 (11)	0.0473 (4)	
C36	0.8020 (2)	0.3417 (2)	−0.02921 (13)	0.0574 (5)	
H36A	0.7843	0.3453	−0.0819	0.069*	
C37	0.7069 (2)	0.3415 (2)	0.03854 (14)	0.0589 (5)	
H37A	0.6232	0.3465	0.0328	0.071*	
C38	0.7372 (2)	0.3336 (2)	0.11663 (13)	0.0534 (5)	
H38A	0.6713	0.3343	0.1626	0.064*	
C39	0.94879 (19)	0.32743 (16)	0.06202 (10)	0.0410 (4)	
C40	1.07354 (18)	0.32791 (16)	0.07513 (10)	0.0412 (4)	

### Atomic displacement parameters (Å^2^)

**Table:**

	*U*^11^	*U*^22^	*U*^33^	*U*^12^	*U*^13^	*U*^23^
Cd	0.04353 (8)	0.03857 (8)	0.02888 (7)	−0.00945 (6)	−0.00605 (5)	−0.00080 (5)
N1	0.0494 (8)	0.0401 (8)	0.0362 (7)	−0.0122 (6)	−0.0113 (6)	−0.0016 (6)
N2	0.0510 (9)	0.0438 (8)	0.0333 (6)	−0.0173 (7)	−0.0040 (6)	−0.0008 (6)
N3	0.0524 (9)	0.0436 (9)	0.0339 (7)	−0.0165 (7)	−0.0077 (6)	0.0043 (6)
N4	0.0493 (8)	0.0443 (9)	0.0345 (7)	−0.0131 (7)	−0.0044 (6)	−0.0035 (6)
O1	0.0432 (7)	0.0480 (8)	0.0589 (8)	−0.0097 (6)	−0.0054 (6)	−0.0065 (6)
O1W	0.0756 (12)	0.0716 (12)	0.0743 (11)	0.0018 (9)	−0.0069 (9)	−0.0048 (9)
O2	0.0553 (8)	0.0398 (6)	0.0501 (7)	−0.0133 (6)	−0.0174 (6)	−0.0004 (5)
O2W	0.0627 (10)	0.0828 (12)	0.0755 (11)	−0.0294 (9)	−0.0132 (8)	0.0067 (9)
O3	0.0609 (9)	0.0822 (11)	0.0429 (8)	−0.0134 (8)	−0.0043 (7)	−0.0031 (7)
O3W	0.0661 (11)	0.0913 (14)	0.1104 (15)	−0.0253 (10)	−0.0219 (10)	0.0169 (12)
O4	0.0601 (9)	0.0574 (9)	0.0463 (7)	−0.0142 (7)	−0.0026 (6)	−0.0064 (6)
O4W	0.0813 (13)	0.0856 (14)	0.1083 (16)	−0.0318 (11)	−0.0225 (11)	0.0170 (11)
O5	0.0506 (8)	0.0453 (7)	0.0575 (8)	−0.0149 (6)	−0.0145 (6)	0.0001 (6)
O5W	0.0835 (14)	0.0992 (16)	0.166 (2)	−0.0272 (12)	−0.0632 (15)	0.0193 (15)
O6	0.0626 (10)	0.0939 (13)	0.0498 (8)	−0.0186 (9)	−0.0159 (7)	−0.0048 (8)
C1	0.0549 (11)	0.0462 (11)	0.0346 (8)	−0.0199 (9)	−0.0114 (8)	0.0077 (7)
C2	0.0693 (14)	0.0537 (12)	0.0538 (11)	−0.0328 (11)	−0.0165 (10)	0.0113 (9)
C3	0.0566 (11)	0.0387 (10)	0.0523 (11)	−0.0222 (9)	−0.0142 (9)	0.0016 (8)
C4	0.0509 (11)	0.0451 (11)	0.0645 (13)	−0.0069 (9)	−0.0192 (10)	−0.0028 (9)
C5	0.0486 (11)	0.0544 (12)	0.0572 (12)	−0.0065 (9)	−0.0068 (9)	−0.0109 (9)
C6	0.0512 (11)	0.0476 (11)	0.0505 (11)	−0.0152 (9)	−0.0120 (9)	−0.0050 (8)
C7	0.0447 (10)	0.0552 (12)	0.0577 (12)	−0.0141 (9)	−0.0133 (9)	−0.0028 (9)
C8	0.0486 (11)	0.0561 (12)	0.0542 (11)	−0.0194 (9)	−0.0049 (9)	−0.0042 (9)
C9	0.0485 (10)	0.0375 (9)	0.0323 (8)	−0.0136 (8)	−0.0035 (7)	0.0026 (7)
C10	0.0509 (11)	0.0354 (9)	0.0475 (10)	−0.0136 (8)	−0.0038 (8)	0.0008 (7)
C11	0.0437 (9)	0.0335 (9)	0.0457 (9)	−0.0128 (7)	−0.0046 (7)	−0.0054 (7)
C12	0.0474 (11)	0.0693 (14)	0.0483 (11)	−0.0016 (10)	−0.0118 (9)	0.0016 (10)
C13	0.0414 (11)	0.0802 (16)	0.0521 (12)	0.0001 (10)	−0.0032 (9)	−0.0027 (11)
C14	0.0495 (11)	0.0499 (11)	0.0446 (10)	−0.0134 (9)	−0.0060 (8)	−0.0060 (8)
C15	0.0491 (11)	0.0541 (12)	0.0504 (11)	−0.0133 (9)	−0.0166 (9)	−0.0021 (9)
C16	0.0384 (9)	0.0481 (11)	0.0563 (11)	−0.0094 (8)	−0.0091 (8)	−0.0075 (9)
C17	0.0549 (12)	0.0485 (11)	0.0499 (11)	−0.0088 (9)	−0.0107 (9)	−0.0052 (9)
C18	0.0547 (13)	0.0549 (14)	0.0797 (16)	−0.0058 (10)	−0.0165 (11)	−0.0023 (11)
C19	0.0658 (14)	0.0528 (13)	0.0791 (16)	−0.0124 (11)	−0.0369 (12)	0.0115 (11)
C20	0.0696 (13)	0.0458 (11)	0.0511 (11)	−0.0262 (10)	−0.0290 (10)	0.0087 (8)
C21	0.0953 (19)	0.0643 (14)	0.0533 (12)	−0.0381 (14)	−0.0414 (13)	0.0193 (11)
C22	0.105 (2)	0.0755 (16)	0.0346 (10)	−0.0518 (15)	−0.0259 (11)	0.0135 (10)
C23	0.0796 (14)	0.0577 (12)	0.0328 (9)	−0.0408 (11)	−0.0105 (9)	0.0011 (8)
C24	0.0872 (17)	0.0794 (16)	0.0338 (9)	−0.0463 (14)	0.0050 (10)	−0.0082 (10)
C25	0.0656 (14)	0.0715 (15)	0.0514 (12)	−0.0265 (12)	0.0107 (10)	−0.0152 (11)
C26	0.0553 (12)	0.0559 (12)	0.0477 (11)	−0.0157 (10)	0.0001 (9)	−0.0029 (9)
C27	0.0593 (11)	0.0411 (10)	0.0320 (8)	−0.0268 (9)	−0.0102 (7)	0.0008 (7)
C28	0.0556 (11)	0.0366 (9)	0.0386 (9)	−0.0221 (8)	−0.0176 (8)	0.0023 (7)
C29	0.0537 (12)	0.0532 (12)	0.0479 (10)	−0.0136 (9)	−0.0094 (9)	−0.0066 (9)
C30	0.0481 (11)	0.0593 (13)	0.0664 (14)	−0.0163 (10)	−0.0032 (10)	−0.0105 (10)
C31	0.0539 (12)	0.0534 (13)	0.0562 (12)	−0.0139 (10)	0.0088 (9)	−0.0054 (9)
C32	0.0533 (11)	0.0388 (10)	0.0421 (9)	−0.0092 (8)	0.0028 (8)	−0.0027 (7)
C33	0.0724 (15)	0.0553 (13)	0.0341 (9)	−0.0137 (11)	0.0068 (9)	0.0014 (8)
C34	0.0755 (15)	0.0580 (13)	0.0307 (9)	−0.0134 (11)	−0.0062 (9)	0.0018 (8)
C35	0.0659 (12)	0.0369 (10)	0.0351 (9)	−0.0111 (9)	−0.0106 (8)	0.0005 (7)
C36	0.0780 (15)	0.0534 (12)	0.0458 (11)	−0.0212 (11)	−0.0245 (10)	0.0051 (9)
C37	0.0640 (13)	0.0610 (14)	0.0603 (13)	−0.0263 (11)	−0.0245 (11)	0.0117 (10)
C38	0.0568 (12)	0.0558 (12)	0.0493 (11)	−0.0223 (10)	−0.0109 (9)	0.0098 (9)
C39	0.0549 (11)	0.0314 (9)	0.0329 (8)	−0.0105 (8)	−0.0062 (7)	0.0000 (6)
C40	0.0512 (10)	0.0321 (9)	0.0331 (8)	−0.0080 (8)	−0.0015 (7)	−0.0025 (6)

### Geometric parameters (Å, °)

**Table:**

Cd—O2	2.3221 (13)	C10—H10B	0.9700
Cd—N2	2.3609 (14)	C11—C12	1.374 (3)
Cd—O5	2.3672 (14)	C11—C16	1.386 (3)
Cd—N4	2.3790 (15)	C12—C13	1.384 (3)
Cd—N3	2.4447 (15)	C12—H12A	0.9300
Cd—N1	2.4522 (15)	C13—C14	1.375 (3)
Cd—O1	2.7419 (14)	C13—H13A	0.9300
Cd—O4	2.8591 (15)	C14—C15	1.378 (3)
N1—C17	1.325 (2)	C15—C16	1.381 (3)
N1—C28	1.354 (2)	C15—H15A	0.9300
N2—C26	1.320 (3)	C16—H16A	0.9300
N2—C27	1.352 (2)	C17—C18	1.393 (3)
N3—C38	1.322 (3)	C17—H17A	0.9300
N3—C39	1.353 (2)	C18—C19	1.357 (3)
N4—C29	1.320 (3)	C18—H18A	0.9300
N4—C40	1.356 (2)	C19—C20	1.391 (3)
O1—C9	1.242 (2)	C19—H19A	0.9300
O1W—H1WA	0.8283	C20—C28	1.410 (3)
O1W—H1WB	0.7668	C20—C21	1.432 (3)
O2—C9	1.265 (2)	C21—C22	1.331 (4)
O2W—H2WA	0.8134	C21—H21A	0.9300
O2W—H2WB	0.7638	C22—C23	1.426 (3)
O3—C14	1.372 (2)	C22—H22A	0.9300
O3—H3A	0.8200	C23—C24	1.400 (3)
O3W—H3WA	0.8409	C23—C27	1.413 (2)
O3W—H3WB	0.8210	C24—C25	1.354 (4)
O4—C1	1.248 (2)	C24—H24A	0.9300
O4W—H4WA	0.8194	C25—C26	1.399 (3)
O4W—H4WB	0.8719	C25—H25A	0.9300
O5—C1	1.256 (2)	C26—H26A	0.9300
O5W—H5WB	0.8405	C27—C28	1.442 (3)
O5W—H5WA	0.9152	C29—C30	1.388 (3)
O6—C6	1.366 (2)	C29—H29A	0.9300
O6—H6A	0.8200	C30—C31	1.364 (3)
C1—C2	1.511 (3)	C30—H30A	0.9300
C2—C3	1.519 (3)	C31—C32	1.395 (3)
C2—H2A	0.9700	C31—H31A	0.9300
C2—H2B	0.9700	C32—C40	1.407 (2)
C3—C8	1.385 (3)	C32—C33	1.434 (3)
C3—C4	1.388 (3)	C33—C34	1.338 (3)
C4—C5	1.374 (3)	C33—H33A	0.9300
C4—H4A	0.9300	C34—C35	1.426 (3)
C5—C6	1.381 (3)	C34—H34A	0.9300
C5—H5A	0.9300	C35—C36	1.395 (3)
C6—C7	1.380 (3)	C35—C39	1.413 (2)
C7—C8	1.386 (3)	C36—C37	1.361 (3)
C7—H7A	0.9300	C36—H36A	0.9300
C8—H8A	0.9300	C37—C38	1.393 (3)
C9—C10	1.519 (3)	C37—H37A	0.9300
C10—C11	1.515 (2)	C38—H38A	0.9300
C10—H10A	0.9700	C39—C40	1.439 (3)
			
O2—Cd—N2	80.97 (5)	C16—C11—C10	121.53 (17)
O2—Cd—O5	139.64 (5)	C11—C12—C13	121.49 (19)
N2—Cd—O5	92.60 (5)	C11—C12—H12A	119.3
O2—Cd—N4	99.31 (5)	C13—C12—H12A	119.3
N2—Cd—N4	143.65 (5)	C14—C13—C12	120.21 (19)
O5—Cd—N4	108.46 (5)	C14—C13—H13A	119.9
O2—Cd—N3	83.91 (5)	C12—C13—H13A	119.9
N2—Cd—N3	146.00 (5)	O3—C14—C13	122.12 (18)
O5—Cd—N3	79.64 (5)	O3—C14—C15	118.63 (18)
N4—Cd—N3	68.89 (5)	C13—C14—C15	119.24 (18)
O2—Cd—N1	133.16 (5)	C14—C15—C16	119.94 (18)
N2—Cd—N1	69.41 (5)	C14—C15—H15A	120.0
O5—Cd—N1	78.78 (5)	C16—C15—H15A	120.0
N4—Cd—N1	85.63 (5)	C15—C16—C11	121.53 (18)
N3—Cd—N1	139.01 (5)	C15—C16—H16A	119.2
O2—Cd—O1	50.72 (4)	C11—C16—H16A	119.2
N2—Cd—O1	77.84 (5)	N1—C17—C18	123.1 (2)
O5—Cd—O1	165.14 (5)	N1—C17—H17A	118.4
N4—Cd—O1	74.83 (5)	C18—C17—H17A	118.4
N3—Cd—O1	114.60 (5)	C19—C18—C17	118.8 (2)
N1—Cd—O1	87.15 (5)	C19—C18—H18A	120.6
O2—Cd—O4	91.80 (5)	C17—C18—H18A	120.6
N2—Cd—O4	74.21 (5)	C18—C19—C20	120.3 (2)
O5—Cd—O4	48.54 (4)	C18—C19—H19A	119.9
N4—Cd—O4	141.63 (5)	C20—C19—H19A	119.9
N3—Cd—O4	76.03 (5)	C19—C20—C28	117.51 (19)
N1—Cd—O4	112.79 (5)	C19—C20—C21	123.2 (2)
O1—Cd—O4	136.30 (4)	C28—C20—C21	119.2 (2)
C17—N1—C28	118.24 (16)	C22—C21—C20	121.5 (2)
C17—N1—Cd	126.59 (13)	C22—C21—H21A	119.3
C28—N1—Cd	115.02 (12)	C20—C21—H21A	119.3
C26—N2—C27	118.82 (16)	C21—C22—C23	121.40 (19)
C26—N2—Cd	123.00 (13)	C21—C22—H22A	119.3
C27—N2—Cd	118.16 (12)	C23—C22—H22A	119.3
C38—N3—C39	117.93 (16)	C24—C23—C27	117.1 (2)
C38—N3—Cd	126.23 (13)	C24—C23—C22	123.47 (19)
C39—N3—Cd	115.81 (12)	C27—C23—C22	119.4 (2)
C29—N4—C40	118.20 (16)	C25—C24—C23	120.35 (19)
C29—N4—Cd	123.74 (13)	C25—C24—H24A	119.8
C40—N4—Cd	118.00 (12)	C23—C24—H24A	119.8
C9—O1—Cd	83.41 (11)	C24—C25—C26	118.9 (2)
H1WA—O1W—H1WB	107.2	C24—C25—H25A	120.6
C9—O2—Cd	102.66 (11)	C26—C25—H25A	120.6
H2WA—O2W—H2WB	117.5	N2—C26—C25	122.8 (2)
C14—O3—H3A	109.5	N2—C26—H26A	118.6
H3WA—O3W—H3WB	106.8	C25—C26—H26A	118.6
C1—O4—Cd	82.56 (12)	N2—C27—C23	122.01 (18)
H4WA—O4W—H4WB	104.7	N2—C27—C28	118.77 (15)
C1—O5—Cd	105.88 (12)	C23—C27—C28	119.20 (18)
H5WB—O5W—H5WA	97.6	N1—C28—C20	122.03 (18)
C6—O6—H6A	109.5	N1—C28—C27	118.64 (16)
O4—C1—O5	122.44 (18)	C20—C28—C27	119.31 (17)
O4—C1—C2	119.67 (19)	N4—C29—C30	123.4 (2)
O5—C1—C2	117.82 (18)	N4—C29—H29A	118.3
C1—C2—C3	110.21 (16)	C30—C29—H29A	118.3
C1—C2—H2A	109.6	C31—C30—C29	118.8 (2)
C3—C2—H2A	109.6	C31—C30—H30A	120.6
C1—C2—H2B	109.6	C29—C30—H30A	120.6
C3—C2—H2B	109.6	C30—C31—C32	120.01 (19)
H2A—C2—H2B	108.1	C30—C31—H31A	120.0
C8—C3—C4	117.58 (19)	C32—C31—H31A	120.0
C8—C3—C2	121.69 (19)	C31—C32—C40	117.40 (18)
C4—C3—C2	120.70 (19)	C31—C32—C33	123.10 (19)
C5—C4—C3	121.46 (19)	C40—C32—C33	119.5 (2)
C5—C4—H4A	119.3	C34—C33—C32	121.14 (19)
C3—C4—H4A	119.3	C34—C33—H33A	119.4
C4—C5—C6	120.3 (2)	C32—C33—H33A	119.4
C4—C5—H5A	119.9	C33—C34—C35	121.35 (19)
C6—C5—H5A	119.9	C33—C34—H34A	119.3
O6—C6—C7	121.77 (19)	C35—C34—H34A	119.3
O6—C6—C5	118.83 (19)	C36—C35—C39	117.69 (18)
C7—C6—C5	119.40 (19)	C36—C35—C34	122.86 (18)
C6—C7—C8	119.80 (19)	C39—C35—C34	119.3 (2)
C6—C7—H7A	120.1	C37—C36—C35	119.80 (19)
C8—C7—H7A	120.1	C37—C36—H36A	120.1
C3—C8—C7	121.48 (19)	C35—C36—H36A	120.1
C3—C8—H8A	119.3	C36—C37—C38	118.8 (2)
C7—C8—H8A	119.3	C36—C37—H37A	120.6
O1—C9—O2	122.91 (17)	C38—C37—H37A	120.6
O1—C9—C10	119.66 (17)	N3—C38—C37	123.7 (2)
O2—C9—C10	117.40 (16)	N3—C38—H38A	118.2
C11—C10—C9	111.22 (15)	C37—C38—H38A	118.2
C11—C10—H10A	109.4	N3—C39—C35	122.14 (18)
C9—C10—H10A	109.4	N3—C39—C40	118.36 (15)
C11—C10—H10B	109.4	C35—C39—C40	119.41 (17)
C9—C10—H10B	109.4	N4—C40—C32	122.17 (18)
H10A—C10—H10B	108.0	N4—C40—C39	118.49 (15)
C12—C11—C16	117.57 (18)	C32—C40—C39	119.27 (16)
C12—C11—C10	120.90 (17)		

### Hydrogen-bond geometry (Å, °)

**Table:**

Cg5 is the centroid of the C3–C8 ring.

**Table:**

*D*—H···*A*	*D*—H	H···*A*	*D*···*A*	*D*—H···*A*
O3—H3A···O4^i^	0.82	1.82	2.641 (2)	175
O6—H6A···O4W^ii^	0.82	1.87	2.670 (3)	164
O1W—H1WA···O5^iii^	0.83	1.93	2.756 (2)	177
O1W—H1WB···O6^ii^	0.77	2.03	2.798 (3)	177
O2W—H2WA···O1^iv^	0.81	2.01	2.812 (2)	168
O2W—H2WB···O1W	0.76	2.04	2.769 (3)	160
O3W—H3WA···O3^i^	0.84	2.02	2.817 (3)	158
O3W—H3WB···O2W	0.82	1.94	2.712 (3)	155
O4W—H4WB···O5W	0.87	1.82	2.682 (3)	168
O5W—H5WB···O2	0.84	1.92	2.756 (2)	176
O5W—H5WA···O3W	0.92	1.87	2.737 (3)	157
O4W—H4WA···Cg5^v^	0.82	2.85	3.583 (2)	151

Symmetry codes: (i) −*x*+1, −*y*+1, −*z*+1; (ii) −*x*+1, −*y*+1, −*z*; (iii) *x*−1, *y*+1, *z*; (iv) *x*−1, *y*, *z*; (v) *x*, *y*+1, *z*.
